# Effect of Colonic Absorption on the Pharmacokinetic Properties of Delayed‐Release and Extended‐Release Methylphenidate: In Vivo, In Vitro, and Modeling Evaluations

**DOI:** 10.1002/cpdd.1089

**Published:** 2022-03-22

**Authors:** Bev Incledon, Chantal Incledon, Roberto Gomeni, Cassandra L. Uchida, Amy Morris, Kim Perry, Jill Kapuscinski

**Affiliations:** ^1^ Ironshore Pharmaceuticals & Development, Inc. Camana Bay Grand Cayman Cayman Islands; ^2^ PharmacoMetrica France La Fouillade France; ^3^ Highland Therapeutics Inc. Toronto Ontario Canada; ^4^ IND 2 Results LLC Atlanta Georgia USA; ^5^ Innovative Analytics Kalamazoo Michigan USA

**Keywords:** attention‐deficit/hyperactivity disorder, colonic absorption, drug delivery, methylphenidate, pharmacokinetics

## Abstract

Most stimulants used to treat attention‐deficit/hyperactivity disorder are administered in the morning and absorbed in the upper gastrointestinal tract. DR/ER‐MPH (formerly HLD200), an evening‐dosed delayed‐release and extended‐release methylphenidate, is predicted to be absorbed in the proximal colon. The pharmacokinetic (PK) profile of DR/ER‐MPH is characterized by an 8‐ to 10‐hour delay in initial methylphenidate absorption and a subsequent gradual increase in plasma concentration, followed by a slow decline. To examine the relationship of absorption site to pharmacokinetics, the DR/ER‐MPH formulation was altered to release methylphenidate in the small intestine and distal colon. The 3 formulations were administered in an open‐label, 3‐way, crossover study in healthy adults (N = 18). Compared with the small intestine formulation, the PK profile of the proximal colon (DR/ER‐MPH) formulation exhibited a longer delay before initial methylphenidate absorption, decreased peak methylphenidate concentration, increased time to peak concentration, and decreased bioavailability; these characteristics were amplified in the distal colon formulation. Safety profiles fell within the expectations for methylphenidate products. Modeled PK profiles were similar between the small intestine formulation and a morning‐dosed extended‐release methylphenidate (both predicted to release methylphenidate in the upper gastrointestinal tract), providing additional evidence that the PK profile of DR/ER‐MPH is shaped by colonic absorption.

Attention‐deficit/hyperactivity disorder (ADHD) is a chronic neurodevelopmental disorder that often affects individuals throughout their life span.^1^ The classic symptoms of inattention, hyperactivity, and impulsivity, as well as associated functional impairments, are experienced by individuals from the time of awakening until bedtime.^1^, ^2^, ^3^, ^4^ Left untreated, these symptoms and functional impairments have a profound effect on individuals with ADHD throughout the waking day, including impaired academic/work performance and impaired social functioning.^5^

Long‐acting stimulant medications are recommended as first‐line therapy for the treatment of ADHD.^6^ Over the past 2 decades, many long‐acting stimulant formulations have been developed with differing drug release profiles and dosage forms.^7^ The majority of long‐acting stimulant formulations are administered in the morning and include a combination of immediate‐release and extended‐release components in varying ratios^7^; as a result, drug release and absorption are predicted to occur primarily in the upper gastrointestinal tract. Absorption in this area of the gastrointestinal tract is highly efficient and facilitates rapid achievement of therapeutic stimulant plasma concentrations via an immediate‐release bolus of drug, which provides efficacy shortly after morning administration (with lag depending on the formulation).^7^, ^8^ However, this rapid absorption is also in part responsible for the rapid waning of therapeutic effect with immediate‐release stimulants, typically within a few hours of administration.^7^ The extended‐release components of long‐acting stimulants counteract this effect by slowing drug release (and therefore absorption), typically resulting in a pharmacokinetic (PK) curve with multiple peaks and troughs in stimulant plasma concentration throughout the day, as a result of immediate‐release and extended‐release processes.^7^

DR/ER‐MPH (formerly HLD200) is a delayed‐release and extended‐release methylphenidate that uses the DELEXIS drug delivery platform (DDDP; Ironshore Pharmaceuticals & Development, Inc., Grand Cayman, Cayman Islands) to delay the release of methylphenidate for 8 to 10 hours, allowing it to be administered in the evening with initial methylphenidate release coinciding with awakening the following morning.^9^, ^10^ The DDDP is composed of proprietary microbeads consisting of a drug‐containing core surrounded by 2 functional film layers. The outer delayed‐release layer has hydrophobic, hygroscopic, and pH‐sensitive properties, which control the time it takes for the layer to wet and allow fluid to access the inner extended‐release layer. The extended‐release layer has hydrophobic and soluble properties, which regulate its permeability and control dissolution from the drug‐containing core. The delayed‐release and extended‐release layers work synergistically to provide a prolonged delay in initial drug release, followed by an extended period of drug release.^9^ Because therapeutic plasma concentrations of methylphenidate are already achieved at the time of awakening, there is no need for an immediate‐release methylphenidate component in DR/ER‐MPH.

The PK profile of DR/ER‐MPH is characterized by the 8‐ to 10‐hour delay in methylphenidate release followed by a period of extended controlled release, resulting in an ascending absorption profile with a single peak in plasma concentration (C_max_) occurring ≈14 hours after administration and an extended elimination phase with ≥50% of methylphenidate absorption occurring after C_max_ is reached.^10^ Gamma scintigraphy studies using multiparticulate pellets of similar size, density, and surface characteristics to DR/ER‐MPH demonstrated that the pellets arrived in the colon 4 to 7 hours after ingestion and remained in the colon for at least 24 hours after ingestion in healthy adults.^11^, ^12^, ^13^ Therefore, in DR/ER‐MPH, methylphenidate is predicted to be released and absorbed in the proximal colon. It is hypothesized that colonic absorption may be a key contributor in shaping the PK profile of DR/ER‐MPH. Compared with the small intestine, the colon has a smaller surface area, a larger luminal volume, a lack of villi, and a thick mucus layer, which results in a lower solute absorptive potential.^8^, ^14^ This is predicted to result in a slower rate, and therefore extended period, of methylphenidate absorption.

The aim of this study was to investigate the effects of absorption site on the PK profile of DR/ER‐MPH by performing in vivo and in vitro analyses of 3 formulations of methylphenidate using the DDDP, designed to release methylphenidate at different locations within the gastrointestinal tract: the small intestine, the proximal colon (DR/ER‐MPH), and the distal colon. Additionally, PK modeling was performed for the small intestine formulation and compared with another extended‐release methylphenidate formulation that is also predicted to be released in the upper gastrointestinal tract to determine whether an intrinsic property of the DDDP or the absorption site as conferred by the DDDP plays an important role in shaping the PK profile of DR/ER‐MPH.

## Methods

### In Vivo Study Design

The study protocol and informed consent form for this phase 1, single‐dose, open‐label, randomized, crossover, comparative bioavailability study of healthy adults were reviewed and approved by an Institutional Review Board (Salus IRB, Austin, Texas) for the investigational site in accordance with the US Food and Drug Administration regulations set forth in 21 Code of Federal Regulations Part 56. Informed consent was collected from all participants before enrollment. The study was conducted at a single clinical center (Prism Clinical Research, LLC, St. Paul, Minnesota) in compliance with the Declaration of Helsinki and Good Clinical Practice for Trials on Medicinal Products.

Small intestine, proximal colon (DR/ER‐MPH; trade name: JORNAY PM; Ironshore Pharmaceuticals & Development, Inc.), and distal colon formulations of the DDDP containing methylphenidate were developed, which differed only by the amount of the extended‐release layer that was applied to each bead. Eighteen subjects were randomized to receive a single 100‐mg dose of the 3 formulations of the DDDP containing methylphenidate using a 3‐way crossover study design. Subjects were admitted to the clinical research unit by noon on the day they received their first dose (day 1) and remained in the clinical research unit until 3 days after receiving their final dose (day 12). Subjects were dosed at approximately 9:00 pm on days 1, 5, and 9, and received a standard low‐fat meal 3 hours before dosing and a standard breakfast 12 hours after dosing. Each dose was administered with 240 mL of room‐temperature water, and water intake was permitted ad libitum except for a 1‐hour restriction after each drug administration. Capsules were swallowed whole and were not chewed or opened and sprinkled onto food. There was a 4‐day washout period between each dose administration.

### Participants

Eligible participants were healthy men and women aged 18 to 55 years with a body mass index of 18.5 to 30 kg/m^2^ and a body weight of 55 to 85 kg. Inclusion criteria included but were not limited to the following: (1) general good health with no clinically significant abnormal findings at the screening examination; and (2) female subjects had a negative pregnancy test at the screening and day 1 visits, and all subjects agreed to practice a highly effective method of contraception for 90 days after their last dose of the study drug.

Exclusion criteria included but were not limited to the following: (1) history or presence of clinically significant cardiovascular, pulmonary, hepatic, renal, hematologic, gastrointestinal (including narrowing of the gastrointestinal tract), endocrine, immunologic, dermatologic, neurologic, oncologic, or psychiatric disease or any other condition that, in the opinion of the principal investigator, would jeopardize the safety of the subject or the validity of the study results; (2) history of glaucoma; (3) history of allergic reactions to methylphenidate, other stimulants, or the inactive components of the DDDP, or current use of monoamine oxidase inhibitors (ie, within 2 weeks of study entry); (4) history of illicit or prescription drug abuse or alcohol abuse in the past year or current evidence of such abuse or addiction in the opinion of the investigator; (5) history of any condition that may interfere with the absorption, distribution, metabolism, or excretion of the DDDP containing methylphenidate; (6) positive for HIV, hepatitis B, or hepatitis C; and (7) participation in an investigational drug study within the greater of 30 days or 5 half‐lives of the study drug before clinical research unit admission.

Participants were required to abstain from dietary supplements, vitamins, herbal medications, antacids, prescription drugs (other than hormonal birth control), nonprescription drugs taken for nontherapeutic indications, broccoli, brussels sprouts, grapefruit, grapefruit juice, and Seville oranges for 7 days before clinical research unit admission through the end of the study. For 3 days before clinical research unit admission through the end of the study, participants agreed not to consume chocolate‐, alcohol‐, xanthene‐, caffeine‐, or poppy‐containing products. In addition, participants agreed to refrain from strenuous physical activity outside of their normal daily routine for 2 days before clinical research unit admission through the end of the study.

### Sample Preparation and PK Analyses

Blood samples (4 mL) were obtained at the following time points: 5 minutes before dosing and 2, 4, 6, 7, 8, 9, 10, 11, 12, 13, 14, 15, 16, 17, 18, 19, 20, 22, 24, 26, 36, and 48 hours after dosing. Blood samples were collected into prechilled sodium fluoride/potassium oxalate Vacutainer tubes, placed on ice, and then centrifuged at 2113*g* and 4°C for 10 minutes within 60 minutes of collection. The resulting plasma was removed and divided into 2 aliquots, flash frozen, and stored in polypropylene tubes at −70°C within 60 minutes from the start of centrifugation. Samples were batch‐shipped to the analytical laboratory packed on dry ice.

Plasma samples were analyzed for methylphenidate concentrations by using a standardized and validated high‐performance liquid chromatography (HPLC) with tandem mass spectrometry method using methylphenidate‐D_9_ as the internal standard (BioPharma Services, Inc., Toronto, Ontario, Canada). The liquid chromatography system used an Agela Venusil ASB C18 analytical column (Agela Technologies, Torrance, California), 150 mm × 4.6 mm (5‐μm particle size) maintained at 40°C using a mobile phase of acetonitrile/water (70/30), 5 mM ammonium formate, and 1% formic acid at a flow rate of 1.20 mL/min. The analyte and internal standard were detected using an API 4000 mass spectrometer (AB Sciex LLC, Framingham, Massachusetts) equipped with Turbo IonSpray ionization source operated in positive‐ion multiple reaction monitoring mode. The detection used the transitions of protonated molecules at m/z 234.2 → 84.0 for methylphenidate and m/z 243.2 → 93.1 for methylphenidate‐D_9_. Calibration curves were determined for methylphenidate by performing a least squares linear regression (weighted 1/x^2^) on a set of calibration standards. The calibration curves for methylphenidate were linear in the range 0.02 to 20 ng/mL (r^2^ = 0.994). Interassay precision, as measured by the coefficient of variation, for calibration standards ranged from 1.3% to 5.7%. Interassay precision for quality control samples at concentrations of 0.06, 0.16, 10, and 16 ng/mL ranged from 0.4% to 4.7%. Intra‐assay precision was conducted during validation and ranged from 1.0% to 3.4%, as measured by coefficient of variation for quality control samples at concentrations of 0.02, 0.06, 1.6, 10, and 16 ng/mL.

PK parameters were derived from the plasma methylphenidate concentration‐time profiles using actual sampling times and by standard noncompartmental methods in Phoenix WinNonlin, version 7.0 (Certara, Princeton, New Jersey). Missing predose concentrations and values below the limits of quantitation (BLQ; 0.02 ng/mL) before the first quantifiable concentration were set to a concentration value of 0, and missing postdose values or BLQ values after the first quantifiable concentration were set to “missing.” Descriptive statistical analyses were performed using SAS version 9.3 or higher (SAS Institute Inc., Cary, North Carolina).

### Safety Analysis

The safety population was defined as all randomized subjects who provided informed consent and received at least 1 dose of the study drug. Safety was monitored through assessments of spontaneously reported treatment‐emergent adverse events (AEs) in response to a general query and review of clinical data (ie, hematology, biochemistry, urinalysis, body temperature, blood pressure, heart rate, and electrocardiograms). AEs were coded using the Medical Dictionary for Regulatory Affairs, version 21.0. The treatment group attributed to each AE was defined by the last treatment given before AE onset, regardless of the time interval between treatment and AE.

### In Vitro Dissolution

The dissolution profile of each formulation was measured in vitro according to a novel validated dissolution method with HPLC sample analysis. The formulations were tested using a *US Pharmacopeia* General Chapter <711> dissolution Apparatus 1 with 1000‐mL round‐bottom vessels under the following conditions, which simulated conditions in the gastrointestinal tract: stage 1 (0‐2 hours): 700 mL 0.1 N hydrochloric acid (HCl); stage 2 (2‐6 hours): 700 mL 0.1 N HCl and 200 mL 0.2 M sodium phosphate tribasic buffer (pH = 6.0); and stage 3 (6‐24 hours): 700 mL 0.1 N HCl, 200 mL 0.2 M sodium phosphate tribasic buffer, and 10 mL 2 N sodium hydroxide (pH = 7.2). The pH of the selected media would represent physiological conditions of the stomach, upper gastrointestinal tract, and ileum. All stages were performed at 37°C (± 0.5°C) with a basket rotation speed of 75 rpm.

During the dissolution test, 5‐mL aliquots were removed from the dissolution vessels every 2 hours up to 24 hours using a stainless steel cannula or a syringe equipped with a 10‐μm polyethylene full flow filter. From the 5‐mL aliquot, 1 to 2 mL was discarded to waste and the remainder was transferred to a sample tube. Except for the 2‐hour sample, 50 μL of 85% phosphoric acid was added to each sample tube before the addition of the aliquot. Aliquots were allowed to sit for at least 30 minutes before transferring to an HPLC vial.

The aliquots were then analyzed for methylphenidate concentrations using HPLC to calculate the percentage of methylphenidate released using Methylphenidate Hydrochloride *US Pharmacopeia* as an external standard. The liquid chromatography system uses a Zorbax Eclipse XDB‐CN column (Agilent, Santa Clara, California) 150 mm × 4.6 mm (5‐μm particle size) maintained at 30°C and at a flow rate of 2.0 mL/min using a mobile phase of sodium octanesulfonate buffer pH 3.0/acetonitrile (80/20). The sodium octanesulfonate buffer was prepared by dissolving 2.8 g of sodium octanesulfonate in 1000 mL of ultrapurified water and then adjusted to pH 3.0 (± 0.05) using phosphoric acid.

Since methylphenidate is not stable in higher pH dissolution medium, the cumulative percentage of methylphenidate released was calculated by summing methylphenidate hydrochloride and its 2 main degradation products, *erythro* isomer and methylphenidate‐related compound A, to correct for sample solution degradation during dissolution testing. Limits of detection were 0.00031 mg/mL for methylphenidate, 0.00012 mg/mL for *erythro* isomer, and 0.00019 mg/mL for methylphenidate‐related compound A. Limits of quantitation were 0.00094 mg/mL for methylphenidate, 0.00036 mg/mL for *erythro* isomer, and 0.00057 mg/mL for methylphenidate‐related compound A. Missing predose concentrations and BLQ values before the first quantifiable concentration were set to a concentration value of 0, and missing postdose values or BLQ values after the first quantifiable concentration were set to “missing.”

The method was validated over a range of 0.0004 to 0.15 mg/mL of methylphenidate hydrochloride with a correlation coefficient (r) of 0.99991, and over a range of 0.00020 to 0.075 mg/mL of both methylphenidate‐related compound A and *erythro* isomer with a correlation coefficient (r) of 0.999989 and 0.999994, respectively. Accuracy was assessed over the range of concentration for both the lowest (20 mg) and highest (100 mg) strengths and was demonstrated with percent recovery result of 99.1% (range, 98.4%‐101.6%). Intra‐assay precision was assessed through repeatability studies and used percent relative standard deviation (%RSD) criteria, testing 20‐ and 100‐mg strengths. The %RSD at 12 hours was 4.2% and 4.8% for 20 and 100 mg, respectively. The %RSD at 14 hours was 3.3% and 2.7%, respectively. Interassay precision was assessed through intermediate precision studies using absolute difference criteria and testing 20‐ and 100‐mg strengths. Absolute difference at 16 hours was 1.7% and 0.9% and at 20 hours was 1.2% and 1.0% for 20 and 100 mg, respectively. Absolute difference at 12 hours was 0.6% and 0.1%, respectively. Absolute difference at 14 hours was 1.4% and 0.3%, respectively. Robustness studies were executed around filtration, sampling (manual vs automated), mobile phase percent composition, flow rate, and column temperature. No method deviations were found due to the variation in these parameters within the limits tested.

### Comparison of the Small Intestine Formulation to Osmotic Release Oral System Methylphenidate Using PK Modeling

PK data from the in vivo study were used to develop a PK model for the small intestine formulation. A convolution model was used to independently fit the data of the small intestine formulation using a nonlinear mixed‐effect approach, which is an established method for developing population PK models.^15^ As previously described, the concentration‐time profile of DR/ER‐MPH is consistent with a 1‐compartment PK model.^16^ The PK time course of in vivo drug release in the small intestine formulation was best characterized by a sigmoid maximum effect (E_max_)‐type function. Methylphenidate plasma concentration, resulting from an arbitrary dose, was described by convolution as:

(1) $$\frac{dA}{dt}=\;Dose*f\left(t\right)-kel*A$$

where:

(2) $$f\left(t\right)\;=\frac{\mathrm{dr}}{\mathrm{dt}}$$

(3) $$r\left(t\right)\;=\;1-\frac{\mathrm{tim}e^{\mathrm{ga}}}{EC^{\mathrm{ga}}+\mathrm{tim}e^{\mathrm{ga}}}$$

where *A* is the amount of drug, * is the multiplier symbol, *f*(*t*) is the in vivo input function, *kel* is the first‐order elimination rate, *r*(*t*) is the time‐varying fraction of the dose released, *EC* is the time to release 50% of the dose, and *ga* is a parameter characterizing the shape of the absorption curve.

Analyses were conducted in NONMEM version 7.3 (ICON Development Solutions, Dublin, Ireland) using the ADVAN6 subroutine and the first‐order conditional estimation with interaction method. The modeled PK curve for 54‐mg osmotic release oral system methylphenidate (OROS MPH; trade name: CONCERTA, Janssen Pharmaceuticals, Inc., Titusville, New Jersey) was extracted from the literature, which used a similar convolution‐based model but with in vivo release described with a double Weibull function, which most accurately describes its dual release processes.^17^ Comparisons were conducted assuming that the small intestine formulation was administered 4 hours before OROS MPH to account for the delayed release of the DDDP, that is, the small intestine formulation was modeled to be administered at *t* = −4 hours, while OROS MPH was modeled to be administered at *t* = 0 hours, thereby synchronizing the time of initial drug release for both formulations to *t* = 0 hours.

## Results

### Participant Disposition and Characteristics

Baseline demographics of the safety population are summarized in Table 1. All 18 enrolled subjects completed all study activities, and no subjects discontinued or terminated their studies early.

**Table 1:** Subject Demographics

Characteristic	Safety Population (N = 18)
Sex, n (%)	
Male	10 (55.6)
Female	8 (44.4)
Age, years	
Mean (SD)	37.1 (10.9)
Range: min‐max	19‐53
Race, n (%)^a^	
White	16 (88.9)
Black or African American	3 (16.7)
Ethnicity, n (%)	
Not Hispanic/Latino	16 (88.9)
Hispanic/Latino	2 (11.1)
Weight (kg)	
Mean (SD)	70.6 (6.92)
Range: min‐max	59.7‐82.4
Body mass index (kg/m^2^)	
Mean (SD)	23.7 (1.90)
Range: min‐max	20.4‐26.8

SD, standard deviation.

^a^ Subjects may have reported being of >1 race.

### PK Evaluation

**Figure 1:**
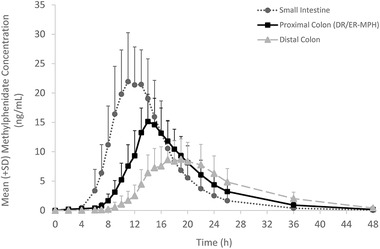
Mean plasma methylphenidate concentrations over time, DR/ER‐MPH, delayed‐release and extended‐release methylphenidate. Error bars indicate SD. Data points were connected with a smoothed line in Excel. SD, standard deviation.

The PK curves of the small intestine, proximal colon (DR/ER‐MPH), and distal colon formulations are illustrated in Figure 1. The small intestine formulation demonstrated a shorter delay before initial methylphenidate absorption compared with the proximal colon (DR/ER‐MPH) formulation, and the distal colon formulation demonstrated a longer delay before initial methylphenidate absorption compared with the proximal colon (DR/ER‐MPH) formulation (Figure 1). Mean methylphenidate concentrations increased at a faster rate and peaked earlier after dosing with the small intestine formulation relative to the proximal colon (DR/ER‐MPH) formulation (Figure 1). Conversely, mean plasma methylphenidate concentrations increased at a slower rate and peaked later after dosing with the distal colon formulation relative to the proximal colon (DR/ER‐MPH) formulation (Figure 1).

PK parameters for the 3 formulations are summarized in Table 2. The PK profile of the small intestine formulation demonstrated increased C_max_, decreased time to peak plasma concentration, and decreased terminal phase half‐life compared with the proximal colon (DR/ER‐MPH) formulation (Figure 1; Table 2). Compared with the proximal colon (DR/ER‐MPH) formulation, the distal colon formulation demonstrated decreased C_max_, increased time to peak plasma concentration, and increased terminal phase half‐life (Figure 1; Table 2). The small intestine formulation demonstrated a 31% increase in methylphenidate bioavailability compared with the proximal colon (DR/ER‐MPH) formulation, and the distal colon formulation demonstrated a 21% decrease in methylphenidate bioavailability compared with the proximal colon (DR/ER‐MPH) formulation (Table 2). The small intestine formulation demonstrated a 63% increase in C_max_ compared with the proximal colon (DR/ER‐MPH) formulation, and the distal colon formulation demonstrated a 41% decrease in C_max_ compared with the proximal colon (DR/ER‐MPH) formulation (Table 2).

**Table 2:** Pharmacokinetic Parameters

	Formulation		
Parameter	Small Intestine (N = 18)	Proximal Colon (DR/ER‐MPH) (N = 18)	Distal Colon (N = 18)
C_max_, ng/mL, mean ± CV (%)	25.2 ± 26.0	15.6 ± 28.8	9.57 ± 38.5
t_max_, h, median (range)	11.0 (8.0‐15.0)	14.0 (13.0‐16.0)	17.0 (14.0‐22.0)
AUC_0‐t_, ng · h/mL, mean ± CV (%)	230 ± 29.0	176 ± 29.7	147 ± 40.9
AUC_0‐∞_, ng · h/mL, mean ± CV (%)	231 ± 28.9	177 ± 30.1	151 ± 41.5
t_1/2_, h, mean (SD)	4.23 (0.566)	4.85 (0.736)	6.05 (0.891)

Relative Bioavailability Parameters, Geometric LS Mean (90%CI)			
	Small Intestine (N = 18)	Proximal Colon (DR/ER‐MPH) (N = 18)	Ratio (Small Intestine:Proximal Colon)
AUC_0‐t_, ng · h/mL	222	169	1.31 (1.18‐1.47)
C_max_, ng/mL	24.4	15.0	1.63 (1.44‐1.84)

	Distal Colon (N = 18)	Proximal Colon (DR/ER‐MPH) (N = 18)	Ratio (Distal Colon:Proximal Colon)
AUC_0‐t_, ng · h/mL	134	169	0.793 (0.710‐0.886)
C_max_, ng/mL	8.81	15.0	0.586 (0.519‐0.663)

AUC_0‐∞_, area under the concentration‐time curve from time 0 (predose) extrapolated to infinite time; AUC_0‐t_, area under the concentration‐time curve from time 0 (predose) to time of last quantifiable concentration; CI, confidence interval; C_max_, peak plasma concentration; CV, coefficient of variation; DR/ER‐MPH, delayed‐release and extended‐release methylphenidate; LS, least squares; SD, standard deviation; t_1/2_, terminal phase half‐life; t_max_, time to peak plasma concentration.

### Safety

AEs are summarized in Table 3. The distal colon formulation was associated with 4 AEs in 3 subjects, while the small intestine and proximal colon (DR/ER‐MPH) formulations were associated with 6 AEs in 4 subjects and 7 AEs in 4 subjects, respectively. All AEs were mild or moderate in severity, no deaths or other serious AEs occurred, and no subjects discontinued the study due to AEs. One subject experienced a single occurrence of an increase in heart rate 12 hours after receiving the small intestine formulation. Single uses of concomitant medication were reported for 3 subjects during the study for the treatment of AEs: acetaminophen was taken by 2 subjects for the treatment of headache, and ondansetron was taken by 1 subject for the treatment of nausea. All reported AEs resolved spontaneously without sequelae by the end of the study. No clinically significant electrocardiogram findings were reported.

**Table 3:** Treatment‐Emergent Adverse Events by System Organ Class

	Formulation		
System Organ Class, n (%) Preferred Term	Small Intestine (N = 18)	Proximal Colon (DR/ER‐MPH) (N = 18)	Distal Colon (N = 18)
Subjects with any AE	4 (22.2)	4 (22.2)	3 (16.7)
Cardiac disorders	1 (5.6)	0	0
Palpitations	1 (5.6)	0	0
Ear and labryinth disorders	1 (5.6)	1 (5.6)	0
Vertigo	1 (5.6)	0	0
Vertigo positional	0	1 (5.6)	0
Gastrointestinal disorders	2 (11.1)	2 (11.1)	2 (11.1)
Dry mouth	1 (5.6)	0	0
Nausea	1 (5.6)	2 (11.1)	2 (11.1)
Injury, poisoning, and procedural complications	0	1 (5.6)	0
Vascular access site hemorrhage	0	1 (5.6)	0
Investigations	1 (5.6)	0	0
Weight decreased	1 (5.6)	0	0
Metabolism and nutrition disorders	0	1 (5.6)	0
Decreased appetite	0	1 (5.6)	0
Nervous system disorders	1 (5.6)	2 (11.1)	1 (5.6)
Headache	0	2 (11.1)	1 (5.6)
Paresthesia	1 (5.6)	0	0
Skin and subcutaneous tissue disorders	0	0	1 (5.6)
Ecchymosis	0	0	1 (5.6)

AE, adverse event; DR/ER‐MPH, delayed‐release and extended‐release methylphenidate.

Subjects who had the same event more than once were counted only once for the preferred term. Subjects who had more than 1 AE within a system organ class were counted only once in that system organ class.

### Dissolution

**Figure 2:**
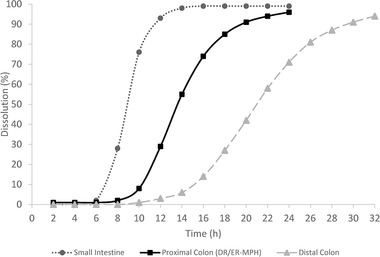
In vitro dissolution. DR/ER‐MPH, delayed‐release and extended‐release methylphenidate. Data points were connected with a smoothed line in Excel.

The dissolution profiles of each formulation are shown in Figure 2. As predicted, dissolution occurred at the fastest rate with the small intestine formulation and at the slowest rate with the distal colon formulation (Figure 2). The time to first observed dissolution was extended as the formulations were designed to release more distally in the gastrointestinal tract; that is, the small intestine formulation began to dissolve at *t* = 6 hours, the proximal colon (DR/ER‐MPH) formulation began to dissolve at *t* = 8 hours, and the distal colon formulation began to dissolve at *t* = 10 hours (Figure 2); these times correlate with the expected sites of release.

### Modeled PK Comparison of the Small Intestine Formulation to OROS MPH

**Figure 3:**
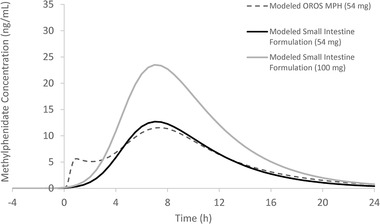
Pharmacokinetic modeling comparison of methylphenidates released in the upper gastrointestinal tract. OROS MPH, osmotic release oral system methylphenidate.

The modeled PK curves of the small intestine formulation and OROS MPH are shown in Figure 3. The PK curves were largely similar in shape, with the exception of the initial peak driven by the immediate‐release methylphenidate component of OROS MPH (between *t* = 0 hours and *t* = 4 hours). The OROS MPH model demonstrated a biphasic profile, with a rapid increase in methylphenidate concentration that peaked at approximately *t* = 1 hour, followed by a short plateau and then a second rise in methylphenidate concentration that peaked at approximately *t* = 7 hours. In contrast, the small intestine model demonstrated a monophasic profile with a single rise in methylphenidate concentration that peaked at approximately *t* = 7 hours (Figure 3). The C_max_ and area under the concentration‐time curve of a 54‐mg small intestine formulation were similar to those of 54‐mg OROS MPH, while the C_max_ and area under the concentration‐time curve of a 100‐mg small intestine formulation were approximately double that of 54‐mg OROS MPH, as expected for formulations with similar absorption and bioavailability (Table 4).

**Table 4:** Estimated Pharmacokinetic Parameters of Modeled OROS MPH (54 mg) and the Small Intestine Formulation (100 mg/54 mg)

Parameter	Modeled Small Intestine Formulation (100 mg)	Modeled Small Intestine Formulation (54 mg)	Modeled OROS MPH (54 mg)
C_max_, ng/mL	23.5	12.7	11.8
AUC_0‐∞_, ng · h/mL	209	113	128
AUC_x‐24_, ng · h/mL	218	118	132

AUC_0‐∞_, area under the concentration‐time curve from time 0 (predose) extrapolated to infinite time; AUC_x‐24_, area under the concentration‐time curve from predose to 24 hours; C_max_, peak plasma concentration; OROS MPH, osmotic release oral system methylphenidate.

## Discussion

The results of these studies demonstrated that the site of methylphenidate absorption within the gastrointestinal tract changes the PK profile of the DDDP containing methylphenidate. Thus, the resulting PK profile of DR/ER‐MPH is the result of delivery conferred by the DDDP that results in absorption in the colon. The small intestine formulation demonstrated a steep ascending curve, high C_max_, and higher bioavailability, while the distal colon formulation demonstrated a very gradual ascending curve, low C_max_, and low bioavailability. The proximal colon (DR/ER‐MPH) formulation produced a PK curve that demonstrated a gradual ascending curve that targeted methylphenidate release in the morning, a dampened C_max_ compared with the small intestine formulation, and reasonable bioavailability that provided methylphenidate exposure throughout the day and into the evening.

The AE profile of each formulation was consistent with the established side effect profile of methylphenidates and of DR/ER‐MPH in particular, and no new safety findings were noted. This indicates that the site of absorption did not affect the tolerability of a single 100‐mg dose of the DDDP containing methylphenidate.

It is noteworthy that the composition and quantity of the delayed‐release layer were identical in all 3 formulations, yet methylphenidate dissolution and release occurred at different times, at different rates, and in areas of the gastrointestinal tract with different pH levels, which indicates that achieving a certain pH threshold is not a trigger for the release of DR/ER‐MPH. Instead, the pH‐sensitive polymer enables dissolution of the delayed‐release layer after it has performed its function of controlling the rate of solvent access to the extended‐release layer; therefore, drug release can still occur without reaching the pH threshold. Rather, the differences in timing of methylphenidate dissolution and release between the small intestine, proximal colon (DR/ER‐MPH), and distal colon formulations are due to the synergistic effects of the delayed‐release and extended‐release layers.

The modeled data presented here demonstrated that the PK curve of the small intestine formulation was largely similar to the modeled OROS MPH PK curve, which is also predicted to be predominantly released and absorbed in the upper gastrointestinal tract. This suggests that the DDDP does not alter the disposition and elimination PK properties of methylphenidate. Thus, the differences in PK parameters between DR/ER‐MPH and other extended‐release methylphenidate formulations are due to colonic absorption, conferred by the DDDP delivering drug to the colon.

Colonic drug delivery has primarily been envisioned as a treatment for local diseases (eg, ulcerative colitis) or as a method to increase bioavailability of a drug via bypass of enzymatic degradation or first‐pass metabolism in the upper gastrointestinal tract.^18^ However, the results of this study show that the benefits of colonic drug delivery can be expanded to include the delivery of medication over an extended period of time (eg, colonic absorption of methylphenidate is absorption rate‐limited, while the small intestinal absorption rate of Biopharmaceutics Classification System class I compounds such as methylphenidate are typically dissolution rate‐limited^19^). Additionally, these data highlight that the DDDP can be modified to optimize PK properties for specific disease conditions by targeting drug absorption to different areas of the gastrointestinal tract and altering duration and extent of drug release.

This study has several limitations. As the study was not designed to evaluate clinical efficacy of the 3 formulations of the DDDP containing methylphenidate, caution should be taken when generalizing the PK findings reported here. Gamma scintigraphic studies of the DDDP are not possible, as radiolabeling the beads would alter their release properties; therefore, the site of methylphenidate absorption for each formulation is predicted by dissolution data. The only difference among the 3 formulations was the amount of the extended‐release layer, which altered the ratio of delayed‐release and extended‐release layer amounts and hence altered the time to methylphenidate release. Therefore, the assumption that differences in the PK profiles are due to the site of absorption is reasonable. Small sample sizes were used; however, this is typical of PK studies. The study was performed in healthy adults rather than in children/adolescents with ADHD; however, a previous study demonstrated that the DR/ER‐MPH PK curves for healthy adults and children/adolescents with ADHD were superimposable when adjusted for body weight.^9^ Finally, subjects received a single 100‐mg dose of the DDDP containing methylphenidate every 4 days, which does not reflect real‐world use of DR/ER‐MPH.

Despite these limitations, the data presented here provide evidence that the site of methylphenidate absorption is a key contributor to the PK parameters of DR/ER‐MPH. This study also demonstrates that colonic absorption via the DDDP can be used to deliver medication with a smooth PK profile over an extended period of time from a single dose.

## Conflicts of Interest

B.I., C.I., and J.K. are employees of Ironshore Pharmaceuticals & Development, Inc. R.G. is a paid consultant to Ironshore Pharmaceuticals & Development, Inc.; Sunovion Pharmaceuticals Inc.; Supernus Pharmaceuticals, Inc.; Teva Branded Pharmaceutical Products R&D, Inc.; Tris Pharma; Biomedical Science Institutes, Singapore; Nanomi BV, The Netherlands; Laboratorios Liconsa S.A., Spain; General Hospital Corporation, Boston, Massachusetts; and UCB Biopharma SPRL. C.U. is an employee of Highland Therapeutics Inc., the parent company of Ironshore Pharmaceuticals & Development, Inc. A.M. is an employee of IND 2 Results, LLC; and K.P. is an employee of Innovative Analytics (sub‐contracted by IND 2 Results, LLC), which was funded by Ironshore Pharmaceuticals & Development, Inc. to conduct the in vivo trial. This research was funded by Ironshore Pharmaceuticals & Development, Inc. Roberto Gomeni is an Honorary Fellow of the American College of Clinical Pharmacology.

## Author Contributions

B.I. conceived the research plan; B.I., C.I., R.G., C.U., A.M., K.P., and J.K. wrote and/or edited the manuscript; B.I., C.I., C.U., A.M., K.P., and J.K. designed the research; C.I. oversaw the formulation development and in vitro dissolution; A.M. and J.K. oversaw the execution of the in vivo study; K.P. performed the in vivo data analyses; R.G. created the PK models and performed the modeling analyses.
